# Induced Pluripotent Stem Cell (iPSC) Lines from a Family with Resistant Epileptic Encephalopathy Caused by Compound Heterozygous Mutations in *SZT2* Gene

**DOI:** 10.3390/ijms232113095

**Published:** 2022-10-28

**Authors:** Cecilia Cattelani, Ingrid Battistella, Francesca Di Leva, Giulia Fioravanti, Francesco Benedicenti, Franco Stanzial, Christine Schwienbacher, Francesca Fanelli, Peter P. Pramstaller, Andrew A. Hicks, Luciano Conti, Corrado Corti

**Affiliations:** 1 Institute for Biomedicine, Eurac Research, Affiliated Institute of the University of Lübeck, 39100 Bolzano, Italy; 2 Laboratory of Stem Cell Biology, Centre for Integrative Biology CIBIO, Università degli Studi di Trento, 38123 Trento, Italy; 3 Clinical Genetics Service and South Tyrol Coordination Center for Rare Diseases, Department of Pediatrics, Regional Hospital of Bolzano, 39100 Bolzano, Italy; 4 Cytogenetics Laboratory, Department of Pathology, Regional Hospital of Bolzano, 39100 Bolzano, Italy

**Keywords:** SZT2, human iPSCs, epileptic and developmental encephalopathies, resistant epilepsy, in vitro models, disease models, therapeutic targets, disease mechanisms

## Abstract

Mutations in the *SZT2* gene have been associated with developmental and epileptic encephalopathy-18, a rare severe autosomal recessive neurologic disorder, characterized by psychomotor impairment/intellectual disability, dysmorphic facial features and early onset of refractory seizures. Here we report the generation of the first induced pluripotent stem cell (iPSC) lines from a patient with treatment-resistant epilepsy, carrying compound heterozygous mutations in *SZT2* (Mut1: c.498G>T and Mut2: c.6553C>T), and his healthy heterozygous parents. Peripheral blood mononuclear cells were reprogrammed by a non-integrating Sendai virus-based reprogramming system. The generated human iPSC lines exhibited expression of the main pluripotency markers, the potential to differentiate into all three germ layers and presented a normal karyotype. These lines represent a valuable resource to study neurodevelopmental alterations, and to obtain mature, pathology-relevant neuronal populations as an in vitro model to perform functional assays and test the patient’s responsiveness to novel antiepileptic treatments.

## 1. Introduction

The seizure threshold 2 protein homolog gene (*SZT2*) encodes a large and relatively uncharacterized protein, predominantly expressed in the developing and mature brain, but also found at lower levels in most human peripheral tissues [1]. *Szt2* was originally identified in mice only a decade ago, and its function was there correlated with epilepsy [2]. Concurrently, a separate study postulated a neuroprotective role in resistance to oxidative stress [1]. More recently, SZT2 was shown to localize to lysosomes and assemble together with KPTN, ITFG2 and C12orf66, in a protein complex called KICSTOR [3]. This complex functions as a negative regulator of the mechanistic target of rapamycin complex 1 (mTORC1) signaling pathway, and is required for the inhibition of this pathway under amino acid or glucose deprivation [3,4]. When SZT2 is deficient, KICSTOR does not assemble, and mTORC1 becomes constitutively localized to the lysosomes and persistently activated, thus promoting biogenesis in the cell even during nutrient shortage [3,4]. Moreover, our recent interactome analysis highlighted the possibility of novel functional roles of SZT2 in additional cellular processes that may or may not be directly mediated by mTORC1, including ciliogenesis, endocytosis, mitochondrial function and neurological disorders [5].

Mutations in *SZT2* have been associated with developmental and epileptic encephalopathy-18 (DEE18), a rare severe autosomal recessive neurologic disorder [6,7]. In a previous report, we described two male siblings, born to healthy non-consanguineous parents, presenting early-onset epilepsy, intellectual disability and macrocephaly [8]. The youngest child is more severely affected clinically. He suffers from a profound psychomotor impairment, and his epilepsy, characterized by daily occurring seizures since birth, is resistant to commonly available anticonvulsants. Despite the difference in phenotype severity, both siblings are compound heterozygous for two point mutations in *SZT2* (Mut1: c.6553C>T; Mut2: c.498G>T; reference sequence NM_015284.4), inherited from the father and the mother, respectively [8].

To date, functional studies on SZT2 have mostly been performed in animals or human immortalized cell lines, thus lacking a suitable model that could reflect the cellular context of the human disease. To overcome this, we have generated the first induced pluripotent stem cell (iPSC) line from a patient carrying the abovementioned *SZT2* mutations, and two additional iPSC lines from the heterozygous parents, to be further differentiated into iPSC-derived neuronal cultures, which will allow future analyses on a more pathology-relevant in vitro model.

## 2. Results

**Figure 1:**
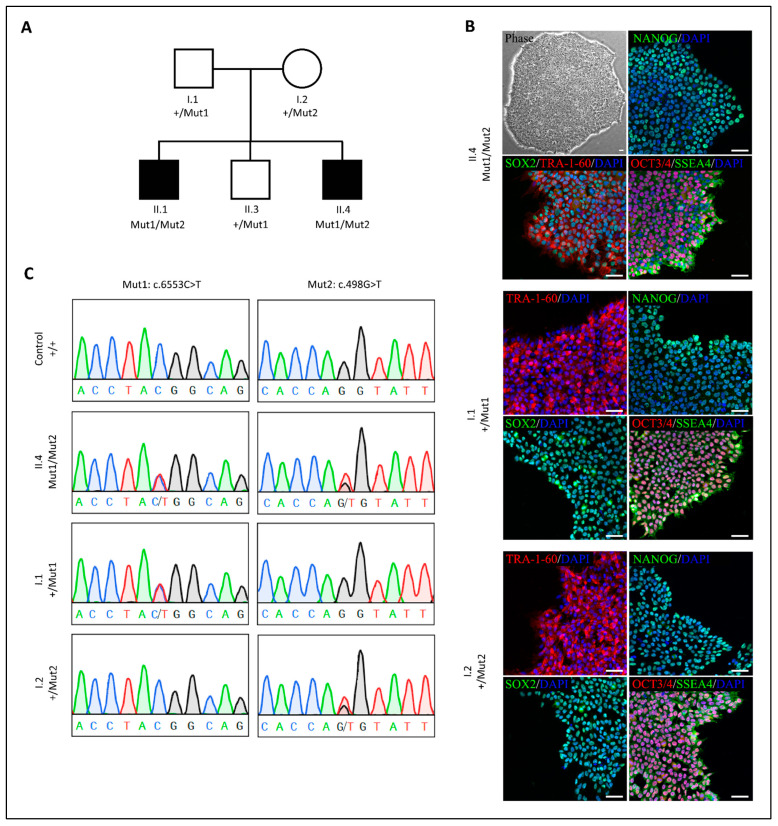
Genotyping and pluripotency characterization of family-derived hiPSCs. (**A**) Family pedigree. Affected individuals are represented by filled black symbols. The (+) sign indicates the reference allele, Mut1 indicates mutation c.6553C>T, and Mut2 indicates mutation c.498G>T. According to an autosomal recessive inheritance pattern, each healthy parent is heterozygous for one of the two identified gene variants. The healthy sibling is a carrier of only one *SZT2* variant (the paternal one), while the affected individuals carry both Mut1 and Mut2. Adapted with permission from Ref. [8]. (**B**) Representative phase microscopy picture of a hiPSC colony from the patient’s line. Immunofluorescence staining of the three cell lines from the family showing the nuclear immunoreactive signal for OCT3/4 (red), SOX2 (green), NANOG (green) and surface localization for SSEA4 (green), TRA-1-60 (red) pluripotency markers. Scale bar 50 μm. (**C**) Sanger sequencing electropherograms from the three subjects’ hiPSC cell lines at the loci of the mutations confirming the expected genotype. The genotype of a control hiPSC line is shown as reference.

To generate human iPSC (hiPSC) lines from the most severely affected patient (II.4) and the parents (I.1, father and I.2, mother) (Figure 1A), peripheral blood mononuclear cells (PBMCs) were reprogrammed by means of the four Yamanaka reprogramming factors (*OCT3/4–SOX2, CMYC, KLF4*) delivered through an integration-free Sendai virus gene-delivery method [9,10,11]. Colonies with hiPSC-like morphology emerged after 10–12 days and were picked 3–4 days later. For each individual, multiple clones with distinct iPSC-like morphology were selected to give rise to stable expanding hiPSC lines, one of which per individual underwent full characterization.

Complete and appropriate reprogramming of the newly generated hiPSC lines was then tested using a range of standard assays, including immunocytochemistry for pluripotency-associated markers and karyotype analysis. Homogeneous and specific expression of the canonical nuclear (NANOG, OCT3/4, SOX2) and surface (SSEA4, TRA-1-60) pluripotency markers was confirmed by immunofluorescence in all hiPSC lines (Figure 1B). The expected genotype at Mut1 (c.6553C>T) and Mut2 (c.498G>T) locus in the *SZT2* gene was verified by Sanger sequencing (Figure 1C).

**Figure 2:**
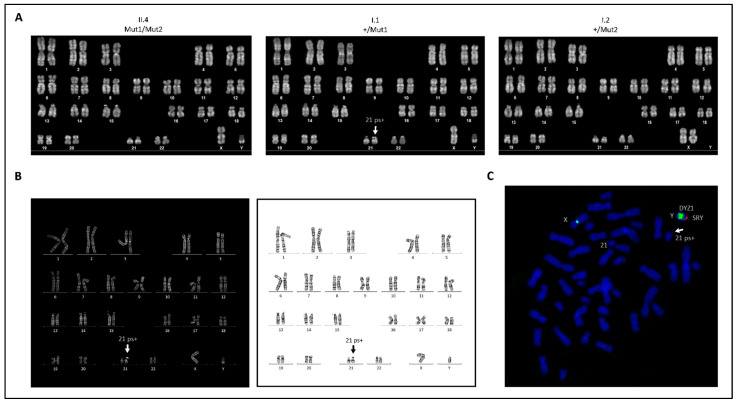
Karyotype analysis. (**A**) Conventional Q-banded karyograms from the hiPSCs clones of the three family members displaying a normal karyotype with no manifest cytogenetic abnormalities, except for an increase in length of the satellite on the short arm of chromosome 21 (21ps+) in the father’s line. (**B**) Conventional Q-banded and G-banded karyograms from the peripheral lymphocytes of the father displaying a normal constitutional karyotype with the 21ps+ heteromorphism. (**C**) SRY-FISH analysis performed on the father’s peripheral lymphocytes showing a normal male (XY) cell with one red signal (SRY; Yp11.31), one green signal (DYZ1; Yq12) and one blue signal (DXZ1; Xp11.1–q11.1). Both 21 chromosomes do not show any Yq-specific green FISH signal. The white arrow indicates the 21ps+ polymorphism. I.1 = father; I.2 = mother; II.4 = patient.

No chromosomal rearrangements resulting from the reprogramming process were detected in any of the three lines (Figure 2A). The patient’s and the mother’s hiPSC lines displayed a normal diploid karyotype (46,XY and 46,XX, respectively) without appreciable abnormalities, whereas the father-derived hiPSC line presented a diploid 46,XY karyotype with an increase in length (+) of the satellite (s) on the short (p) arm of one of the two chromosome 21 (21ps+) (Figure 2A). This polymorphic variant was also found in the constitutional karyotype of the father’s peripheral lymphocytes (Figure 2B) and was predicted to have no consequences on the phenotype [12,13]. Mosaicism was also excluded, as this variant was identified in all 100 analyzed cells. Occasionally, the heteromorphism 21ps+ has been shown to be due to a translocation between the short arm of chromosome 21 and the distal end of the long (q) arm of a Y chromosome. A FISH analysis was performed and confirmed the absence of the Yq/21p translocation (Figure 2C) [12,13].

**Figure 3:**
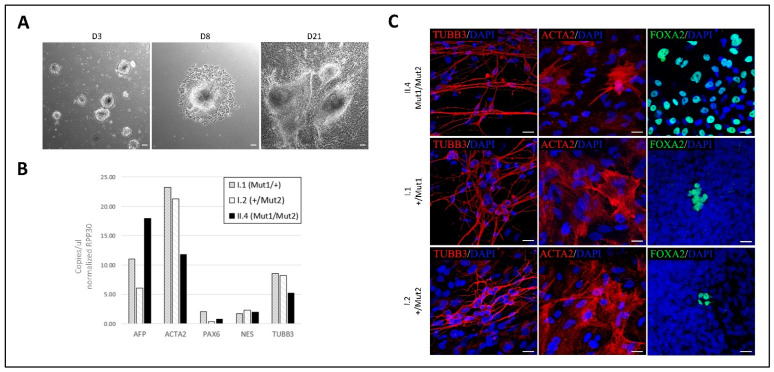
EBs formation assay performed on family-derived iPSCs. (**A**) Representative phase microscopy pictures of three different stages of EBs generation. At day 3 EBs are forming in suspension, at day 7 they are plated in adhesion and allowed to spontaneously differentiate towards the three germ layer derivatives until day 21 when they are processed for downstream analyses. Scale bar 100 μm. (**B**) Droplet digital PCR quantification of ectodermal (*PAX6*, *NES*, *TUBB3*), mesodermal (*ACTA2*) and endodermal (*AFP*) genes mRNA levels. Concentration is expressed as copies/µL and is normalized with the housekeeping gene *RPP30*. Data from one biological replicate. (**C**) Immunofluorescence staining showing the presence of cells expressing specific markers belonging to ectoderm (*TUBB3*) (red), mesoderm (*ACTA2*) (red) and endoderm (*FOXA2*) (green) lineage. Scale bar 20 μm. I.1 = father; I.2 = mother; II.4 = patient.

Pluripotency competence was assessed functionally in vitro by embryoid bodies (EBs) formation assay. Human iPSCs were grown in suspension for one week. At day 7, newly-formed EBs were plated in adhesion and allowed to spontaneously differentiate until day 21, when they were processed for downstream analysis (Figure 3A) [14]. All three hiPSC clones from the family carrying *SZT2* variants were able to give rise to differentiated cells belonging to the three germ layers, as assessed by transcript analysis and immunofluorescence staining. Droplet digital PCR (ddPCR) experiments revealed significant expression of lineage markers representative of endoderm (*AFP*), mesoderm (*ACTA2*) and ectoderm (*PAX6*, *NES*, *TUBB3*) layers (Figure 3B). Confocal microscopy analysis confirmed correct protein expression and localization of typical ectodermal (*TUBB3*), mesodermal (*ACTA2*) and endodermal (*FOXA2*) markers (Figure 3C).

## 3. Discussion

Here, we report the generation of reprogrammed somatic cells from three members of a family carrying pathogenic variants in the *SZT2* gene by using an established Sendai virus-based protocol. The resulting hiPSC clones were able to give rise to stable hiPSC lines, in which we verified the expression of pluripotency-associated markers and the potential of differentiating into derivatives of the three germ layers. Interestingly, our karyotype analysis on the father’s cells revealed the presence of a previously described and well-known heteromorphism on one chromosome 21 [12,13]. This variant (21ps+) consists of an increase in the length of the satellite on the short arm of chromosome 21. The short arms of acrocentric chromosomes, such as chromosome 21, can differ greatly in their length. This is due to the presence of highly variable regions consisting of long stretches of repeated DNA sequences [15,16,17]. Numerous studies have attempted to show a relationship between chromosome 21 short arm variants, such as 21ps+, and an increased risk for non-disjunction, leading to free trisomy 21 in Down syndrome. Similarly, numerous early studies have attempted to show a higher frequency of these variants in parents of Down syndrome children. However, with the advent of banding techniques, it has been shown that there is no difference in the frequency of satellite association in parents of trisomy 21 children [12,13,18,19,20,21]. As no association between the 21ps+ variant and pathological findings has been demonstrated, it is currently believed that this chromosomal variant occurs without phenotypic effect in both carriers and carriers’ offspring. Taken together, the cytogenetic investigations we performed allowed us to establish that the chromosomal variant found in the father-derived hiPSC line is neither a laboratory artifact nor a de novo variant but a clinically harmless constitutional heteromorphism.

Here, we have described the first hiPSC line established from a patient affected by *SZT2*-related epileptic encephalopathy, and his parents. Together, these newly generated cell lines represent a valuable resource to obtain mature, pathology-relevant neuronal populations to be used as an in vitro model to perform molecular, biochemical and electrophysiological studies, aimed at unraveling pathogenic mechanisms leading to DEE18. In addition, considering the pivotal role of mTORC1 in appropriate brain development, these lines can be used to investigate the effect of the two *SZT2* mutations on neurodevelopmental processes, especially corticogenesis. Lastly, as the patient is suffering from an early-onset and drug-resistant form of the disease, this model will serve as a tool to assay responsiveness to novel antiepileptic treatments.

## 4. Materials and Methods

### 4.1. PBMCs Collection and Reprogramming to hiPSCs with Sendai Virus Particles

Blood samples were collected from the most severely affected patient (II.4) and both parents (I.1, father, and I.2, mother) after approval by a local ethics committee and with informed consent from all individual participants or their respective legal guardians (Parere Nr. 26-2019, Comitato Etico dell’Azienda sanitaria di Bolzano). PBMCs from the patient and both parents were isolated in BD Vacutainer^®^ CPT™ cell preparation tubes (362753; Becton Dickinson, Milan, Italy) and separated via centrifuging at room temperature (RT) for 30 min at 1800× *g*. PBMCs were then resuspended in PBS, centrifuged at RT for 15 min at 300× *g* and frozen down in 2 × 10^6^ cells aliquots in fetal bovine serum (FBS) (10270-106; Thermo Fisher Scientific, Monza, Italy) supplemented with 10% dimethyl sulfoxide (DMSO) (D8418; Sigma-Aldrich, Milan, Italy). Upon thawing, cells were centrifuged at 200× *g* for 10 min and cultured in 24 well plates (Costar, Milan, Italy) in PBMC expansion medium with the following composition: StemPro-34 Serum Free Medium (10639011; Thermo Fisher Scientific, Monza, Italy), StemPro-34 Nutrient Supplement (10639011; Thermo Fisher Scientific, Monza, Italy), 200 mM GlutaMAX (35050061; Thermo Fisher Scientific, Monza, Italy), 1% penicillin/streptomycin (15070-063; Thermo Fisher Scientific, Monza, Italy), 100 ng/mL Stem Cell Factor (300-07; Peprotech, DBA, Milan, Italy), 100 ng/mL FLT-3 (300-19; Peprotech, DBA, Milan, Italy), 20 ng/mL interleukin-6 (PHC0065; Thermo Fisher Scientific, Monza, Italy), 20 ng/mL Interleukin-3 (200-03; Peprotech, DBA, Milan, Italy). The medium was replaced daily. At day 4, PBMCs were transduced in feeder-free conditions with Yamanaka’s reprogramming factors (*OCT3/4–SOX2, CMYC, KLF4*) delivered through viral particles provided by the CytoTune-iPS 2.0 Sendai Reprogramming Kit (A16518, Thermo Fisher Scientific, Monza, Italy), as previously reported [22,23]. At day 15 post-infection, optimal hiPSC-like colonies were identified, transferred onto a new well and banked. hiPSCs were cultured as colonies in feeder-free conditions on tissue culture-treated dishes (3516; Costar, Milan, Italy) coated with hESC-Qualified Geltrex solution (A1413301; Thermo Fisher Scientific, Monza, Italy) and maintained in Essential 8 Medium (E8) (A1517001; Thermo Fisher Scientific, Monza, Italy) + 1% penicillin/streptomycin (L0022-100; Biowest, Voden, Monza, Italy). The medium was changed daily, and passaging was performed at confluency (every 3–4 days) via EDTA-based (15575-038; Thermo Fisher Scientific, Monza, Italy) dissociation solution. Cells were maintained in an incubator at 37 °C, 5% CO_2_ and 95% relative humidity.

### 4.2. In Vitro Embryo Bodies Differentiation Assay

For the EBs formation assay, dishes (Costar) were incubated at RT for 1h with 50 mg/mL Pluronic F-127 (P2443-250G; Sigma-Aldrich, Milan, Italy) water solution. iPSC colonies were detached via an EDTA-based (15575-038; Thermo Fisher Scientific, Monza, Italy) dissociation solution and plated in suspension on treated dishes in E8 supplemented with 5μM Y27632 (130-104-169; Miltenyi Biotec, Bologna, Italy). After 48 h, the medium was exchanged with a 1:1 mix of Essential 6 Medium (E6) (A1516401; Thermo Fisher Scientific, Monza, Italy) and E8 medium. At day 4, cultures were shifted to E6, and the medium was changed every other day. At day 7, EBs were collected, plated on Geltrex-coated dishes in E6 to allow growth in adhesion, and allowed to differentiate spontaneously for a further 14 days. The medium was changed every other day and progressively substituted with DMEM High Glucose (L0103-500; Biowest, Voden, Monza, Italy) + 10% FBS (F2442-500ML; Sigma-Aldrich, Milan, Italy). At day 21, EBs were fixed or lysed for further analysis.

### 4.3. RNA Isolation, Retrotranscription and ddPCR

Cell pellets were lysed in RLT buffer supplemented with β-mercaptoethanol (M3148; Sigma-Aldrich, Milan, Italy) and total RNA was extracted using the RNeasy Plus Mini Kit (74136; Qiagen, Milan, Italy), following the manufacturer’s instructions. During RNA purification, on-column digestion of DNA was performed with the RNAse-Free DNase Set (79256; Qiagen, Milan, Italy). Quantification was carried out using the Qubit RNA HS Assay Kit (Q32852, Q32855; Thermo Fisher Scientific, Monza, Italy) and 400ng of RNA was retrotranscribed using the SuperScript VILO cDNA Synthesis Kit (11754-250; Thermo Fisher Scientific, Monza, Italy) following the manufacturer’s protocol. For mRNA expression analysis through ddPCR, predesigned FAM-labelled TaqMan Gene Expression Assays (Thermo Fisher Scientific, Monza, Italy) were employed, according to the manufacturer’s instructions: Alpha-fetoprotein (*AFP*) (Hs00173490_m1), aortic smooth muscle actin (*ACTA2*) (Hs00426835_g1), paired box protein Pax-6 (*PAX6*) (Hs01088114_m1), nestin (*NES*) (Hs04187831_g1), tubulin beta-3 chain (*TUBB3*) (Hs00964962_g1). A HEX-labelled ribonuclease P protein subunit p30 (*RPP30*) ddPCR gene expression assay (dHsaCPE5038241; Bio-Rad, Milan, Italy) was used as an endogenous control. A 20 µL reaction mixture was prepared containing 2X ddPCR Probe Supermix (No dUTP) (1863025; Biorad, Milan, Italy), the primers/probe mix (900 nM primers/250 nM probe) and 1ng of template cDNA. After vortexing and brief spinning at 280 RPM, the solution was loaded into the QX100 droplet generator (Bio-Rad, Milan, Italy) emulsification device and droplets were formed following the manufacturer’s instructions. The contents were transferred to a 96-well reaction plate and sealed with a pre-heated PX1 PCR plate sealer (Bio-Rad, Milan, Italy). Reactions were amplified in a GeneAmp PCR System 9700 thermal cycler (Applied Biosystems, Monza, Italy) with the following conditions: 10 min initial denaturation step at 95 °C, 40 cycles each consisting of a 30 s denaturation at 94 °C followed by an annealing and extension step at 57 °C for 120 s, or 59 °C for 60 s, and a final 10 min extension at 98 °C. Droplets were read immediately with the QX200 droplet reader (Bio-Rad, Milan, Italy) and concentration data for each well were exported from the manufacturer’s software (Bio-Rad QX Manager v. 1.2, Bio-Rad, Milan, Italy) for analysis.

### 4.4. Immunofluorescence Assay

hiPSCs and EBs were seeded onto Geltrex-coated plastic coverslips (20012; SPL Life Sciences, Venice, Italy). Cells were washed twice with PBS, fixed with 4% PFA (158127; Sigma-Aldrich, Milan, Italy) for 30 min, permeabilized with 0.5% Triton X-100 (T8787; Sigma-Aldrich, Milan, Italy) in PBS for 15 min and blocked with 2% Normal Donkey Serum (ab7475; Abcam, Prodotti Gianni, Milan, Italy) + 0.2% Triton X-100 in PBS for 1h, all at RT. Cultures were then incubated with specific primary antibodies (Table 1) in blocking buffer overnight at 4 °C and stained for 1h with secondary antibody (Table 1) in blocking buffer at RT. Nuclei were counterstained using the NucBlue reagent (R37606; Thermo Fisher Scientific; Monza, Italy) for 10 min at RT. To assess the expression of pluripotency genes in hiPSC cultures, the Pluripotent Stem Cell 4-Marker Immunocytochemistry Kit (A24881; Thermo Fisher Scientific, Monza, Italy) was used, following the manufacturer’s protocol. Coverslips were mounted in DAKO fluorescence mounting medium (S3023; Agilent, Milan, Italy) and images were acquired using a Leica SP8-X confocal microscope (Leica Microsystems, Wetzlar, Germany) or a Nikon Eclipse Ti2 fluorescent microscope with a DS-Qi2 camera (Nikon, Firenze, Italy).

### 4.5. Karyotyping

Karyotyping analyses were performed on low passage hiPSCs by the ISENET Biobanking service unit in Milan, Italy (www.isenetbiobanking.com, accessed on 1 September 2021). Cells were treated on the third day after passage with 0.1 μg/mL Colchicine for 3 h. Cytogenetic analysis was carried out using the Q-banding method of at least 20 metaphases with 350 bands resolution. The constitutional chromosomal analysis of the father was performed on lymphocytes isolated from peripheral blood at the Cytogenetics Laboratory of the Regional Hospital of Bolzano. Lymphocyte cell cultures and synchronization were established using the Chromosome Synchro P kit (Euroclone, Milan, Italy) according to the manufacturer’s protocol. Cells were treated after 72 h with 0.1 μg/mL Colchicine for 45 min. Cytogenetic analysis was carried out using the G- and Q- banding method of 25 metaphases with a resolution of 400 bands per haploid set (Figure 2B). An additional 75 cells were controlled to exclude mosaicism for the 21ps+. To exclude an improbable Yq/21p translocation, a FISH analysis was also performed on 20 metaphases from cells at 72 h culture using the Cytocell AQUARIUS SRY probe (LPU026S) according to the manufacturer’s instructions.

### 4.6. PCR and Sequencing

For confirmation of mutation genotype, genomic DNA (gDNA) from iPSC clones was extracted using the QIAamp DNA Mini Kit (Qiagen, Milan, Italy) according to the manufacturer’s protocol. Quantification was carried out using a NanoDrop 1000 spectrophotometer (Thermo Fisher Scientific, Monza, Italy) and quality was checked by running 100–200 ng of each sample on a 1% agarose gel. A 20 µL reaction mixture was prepared containing, AmpliTaq Gold DNA polymerase (5 U/µL) (N8080241; Thermo Fisher Scientific, Monza, Italy), with 10X PCR Buffer II, 25 mM MgCl_2_, 10 mM dNTP Mix, and 10 µM primers listed in Table 2 and 10 ng of template gDNA. Reactions were amplified in a Mastercycler Pro S thermal cycler (Eppendorf, Milan, Italy) using the following conditions: 10 min initial denaturation at 95 °C, 35 cycles each consisting of 10 s denaturation at 95 °C, 30 s annealing at 58 °C and 30 s extension at 72 °C, with a final extension at 72 °C for 5 min. Reactions were run on a 1% agarose gel and bands corresponding to the expected size (Mut 1 = 385 bp fragment; Mut 2 = 372 bp fragment) were excised with a clean scalpel and extracted using a QIAquick PCR & Gel Cleanup Kit (Qiagen, Milan, Italy) according to the manufacturer’s instructions. Sequencing of the PCR products was performed using the Sanger method at Eurofins Genomics (Germany, https://www.eurofins.com, accessed on 12 May 2021) with the forward primers listed in Table 2.

### 4.7. Mycoplasma Test

Cells were routinely tested for the absence of mycoplasma contamination using a MycoAlert Mycoplasma Detection Kit (LT07-518; Lonza, Euroclone, Milan, Italy), following the manufacturer’s instructions.

## Figures and Tables

**Table 1:** Antibodies used for immunocytochemistry.

Antibody	Species	Dilution	Source	Identifier
OCT3/4 ^1^	Rabbit	1:200	Thermo Fisher Scientific	Cat A24867 RRID: AB_2650999
SOX2 ^1^	Rat	1:100	Thermo Fisher Scientific	Cat A24759 RRID: AB_2651000
TRA-1-60 ^1^	Mouse	1:100	Thermo Fisher Scientific	Cat A24868 RRID: AB_2651002
SSEA4 ^1^	Mouse	1:100	Thermo Fisher Scientific	Cat A24866 RRID: AB_2651001
NANOG	Rabbit	1:400	Cell Signaling Technology	Cat 4903 RRID: AB_10559205
FOXA2/HNF3β	Goat	1:100	R&D Biotechne	Cat AF2400 RRID: AB_2294104
αSMA	Mouse	1:1000	Abcam	Cat ab7817 RRID: AB_262054
βIII Tubulin	Mouse	1:5000	Biolegend	Cat 801201 RRID: AB_2313773
Alexa Fluor 488 Donkey anti-Rabbit IgG		1:1000	Thermo Fisher Scientific	Cat A21206 RRID: AB_2535792
Alexa Fluor 555 Donkey anti-Mouse IgG		1:1000	Thermo Fisher Scientific	Cat A31570 RRID: AB_2536180
Alexa Fluor 555 Donkey anti-Rabbit ^1^		1:250	Thermo Fisher Scientific	Cat A24869 RRID: AB_2651006
Alexa Fluor 488 Goat anti-Mouse IgG3 ^1^		1:250	Thermo Fisher Scientific	Cat A24877 RRID: AB_2651008
Alexa Fluor 488 Donkey anti-Rat ^1^		1:250	Thermo Fisher Scientific	Cat A24876 RRID: AB_2651007
Alexa Fluor 555 Goat anti-Mouse IgM ^1^		1:250	Thermo Fisher Scientific	Cat A24871 RRID: AB_2651009

^1^ From Pluripotent Stem Cell 4-Marker Immunocytochemistry Kit.

**Table 2:** Primers used for PCR and sequencing.

Name	Sequence	Target
SZT2 gDNA M1 F	GGAACTGCAAGCTGACACCA	SZT2 genomic Exon 47
SZT2 gDNA M1 R	TCGGTAGGAAAGAGTAAGTGCG	SZT2 genomic Exon 47
SZT2 gDNA M2 F	TGGAAGCTGCATGTTTCTGC	SZT2 genomic Exon 4
SZT2 gDNA M2 R	AACAGGTTCAAGAAGCCAGCA	SZT2 genomic Exon 4

## Data Availability

Not applicable.

## References

[1] Toutzaris D., Lewerenz J., Albrecht P., Jensen L.T., Letz J., Geerts A., Golz S., Methner A. (2010). A novel giant peroxisomal superoxide dismutase motif-containing protein. Free Radic. Biol. Med..

[2] Frankel W.N., Yang Y., Mahaffey C.L., Beyer B.J., O’Brien T.P. (2009). Szt2, a novel gene for seizure threshold in mice. Genes Brain Behav..

[3] Wolfson R.L., Chantranupong L., Wyant G.A., Gu X., Orozco J.M., Shen K., Condon K.J., Petri S., Kedir J., Scaria S.M. (2017). KICSTOR recruits GATOR1 to the lysosome and is necessary for nutrients to regulate mTORC1. Nature.

[4] Peng M., Yin N., Li M.O. (2017). SZT2 dictates GATOR control of mTORC1 signalling. Nature.

[5] Cattelani C., Lesiak D., Liebscher G., Singer I.I., Stasyk T., Wallnöfer M.H., Heberle A.M., Corti C., Hess M.W., Pfaller K. (2021). The SZT2 interactome unravels new functions of the KICSTOR complex. Cells.

[6] Basel-Vanagaite L., Hershkovitz T., Heyman E., Raspall-Chaure M., Kakar N., Smirin-Yosef P., Vila-Pueyo M., Kornreich L., Thiele H., Bode H. (2013). Biallelic SZT2 mutations cause infantile encephalopathy with epilepsy and dysmorphic corpus callosum. Am. J. Hum. Genet..

[7] Trivisano M., Rivera M., Terracciano A., Ciolfi A., Napolitano A., Pepi C., Calabrese C., Digilio M.C., Tartaglia M., Curatolo P. (2020). Developmental and epileptic encephalopathy due to SZT2 genomic variants: Emerging features of a syndromic condition. Epilepsy Behav..

[8] Domingues F.S., König E., Schwienbacher C., Volpato C.B., Picard A., Cantaloni C., Mascalzoni D., Lackner P., Heimbach A., Hoffmann P. (2019). Compound heterozygous SZT2 mutations in two siblings with Early-onset epilepsy, intellectual disability and macrocephaly. Seizure.

[9] Takahashi K., Tanabe K., Ohnuki M., Narita M., Ichisaka T., Tomoda K., Yamanaka S. (2007). Induction of pluripotent stem cells from adult human fibroblasts by defined factors. Cell.

[10] Fusaki N., Ban H., Nishiyama A., Saeki K., Hasegawa M. (2009). Efficient induction of transgene-free human pluripotent stem cells using a vector based on Sendai virus, an RNA virus that does not integrate into the host genome. Proc. Jpn. Acad. Ser. B Phys. Biol. Sci..

[11] Yang W., Mills J.A., Sullivan S., Liu Y., French D.L., Gadue P. (2008). iPSC Reprogramming from human peripheral blood using Sendai virus mediated gene transfer. Stembook.

[12] Barber J.C.K. (2005). Atlas of human chromosome heteromorphisms. Hum. Genet..

[13] Gardner R.J.M., Amor D.J. (2018). Gardner and Sutherland’s Chromosome Abnormalities and Genetic Counseling.

[14] Carpenter M.K., Rosler E., Rao M.S. (2003). Characterization and differentiation of human embryonic stem cells. Cloning Stem Cells.

[15] Gosden J.R., Lawrie S.S., Gosden C.M. (1981). Satellite DNA sequences in the human acrocentric chromosomes: Information from translocations and heteromorphisms. Am. J. Hum. Genet..

[16] Waye J.S., Willard H.F. (1989). Human β satellite DNA: Genomic organization and sequence definition of a class of highly repetitive tandem DNA. Proc. Natl. Acad. Sci. USA.

[17] Redaelli S., Conconi D., Villa N., Sala E., Crosti F., Corti C., Catusi I., Garzo M., Romitti L., Martinoli E. (2020). Instability of short arm of acrocentric chromosomes: Lesson from non-acrocentric satellited chromosomes. Report of 24 Unrelated Cases. Int. J. Mol. Sci..

[18] Cooke P., Curtis D.J. (1974). General and specific patterns of acrocentric association in parents of mongol children. Hum. Genet..

[19] Taysi K. (1975). Satellite association: Giemsa banding studies in parents of Down’s Syndrome patients. Clin. Genet..

[20] Yip M.Y., Fox D.P. (1981). Variation in pattern and frequency of acrocentric association in normal and Trisomy-21 individuals. Hum. Genet..

[21] Jacobs P.A., Mayer M. (1981). The origin of human Trisomy: A study of heteromorphisms and satellite associations. Ann. Hum. Genet..

[22] Marcatili M., Marsoner F., D’Agostino A., Karnavas T., Bottai D., Scarone S., Conti L. (2016). Establishment of an induced pluripotent stem cell (IPSC) line from a patient with clozapine-responder Schizophrenia. Stem Cell Res..

[23] Marsoner F., Marcatili M., Karnavas T., Bottai D., D’Agostino A., Scarone S., Conti L. (2016). Generation and characterization of an induced pluripotent stem cell (IPSC) line from a patient with clozapine-resistant Schizophrenia. Stem Cell Res..

